# Case Report: Long-term control of brain metastases with over 100 cycles of T-DM1 in HER2-positive metastatic breast cancer

**DOI:** 10.3389/fonc.2026.1778267

**Published:** 2026-01-29

**Authors:** Hyemi Ko, Song-Yi Choi, Donghyun Kim, Jinsun Lee

**Affiliations:** 1 Department of Surgery, Chungnam National University Hospital, Daejeon, Republic of Korea; 2 Department of Surgery, Chungnam National University, School of Medicine, Daejeon, Republic of Korea; 3 Department of Pathology, Chungnam National University School of Medicine, Chungnam National University Hospital, Daejeon, Republic of Korea

**Keywords:** antibody-drug conjugate, brain metastasis, case report, HER2-positive breast cancer, long-term therapy, stereotactic radiosurgery, T-DM1, trastuzumab emtansine

## Abstract

Human epidermal growth factor receptor 2 (HER2)-positive breast cancer is associated with an aggressive clinical course and a high incidence of central nervous system (CNS) metastases. Although trastuzumab emtansine (T-DM1) is a standard therapy for previously treated HER2-positive metastatic breast cancer, reports of exceptionally prolonged administration with long-term intracranial stability remain scarce. Here, we describe a patient with HER2-positive metastatic breast cancer with brain metastases who achieved long-term systemic and intracranial stability following CNS-directed local therapy and subsequent T-DM1 treatment. T-DM1 was administered from May 2019 to November 2025 (107 cycles; >6 years), and serial imaging every 3–4 months demonstrated continued disease stability without the development of new metastatic lesions. Long-term tolerability was acceptable, with only intermittent grade 1 hyperbilirubinemia without dose modification and preserved cardiac function on longitudinal monitoring. This case suggests that prolonged administration of T-DM1 may be feasible in carefully selected patients with durable clinical benefit and manageable toxicity, emphasizing the role of individualized treatment decisions, imaging surveillance, and long-term safety monitoring.

## 1 Introduction

Human epidermal growth factor receptor 2 (HER2)-positive breast cancer accounts for approximately 15–20% of breast cancers and is characterized by an aggressive clinical course and a high propensity for central nervous system (CNS) metastases. Despite major advances in HER2-targeted systemic therapies, CNS metastasis remains a frequent and clinically significant complication, particularly as systemic disease control and survival improve (1).

Trastuzumab emtansine (T-DM1) is an antibody–drug conjugate linking trastuzumab to the microtubule inhibitor DM1, and is a standard treatment option for patients with HER2-positive metastatic breast cancer previously treated with trastuzumab and a taxane (2, 3). In the phase III EMILIA trial, T-DM1 significantly improved overall survival compared to capecitabine plus lapatinib, establishing T-DM1 as a standard therapy in this setting (3).

Although patients with active brain metastases are largely excluded from pivotal clinical trials, CNS disease progression remains a clinically relevant challenge to long-term disease control. Data regarding prolonged administration of T-DM1 with sustained intracranial disease stability are limited.

Here, we report an exceptional case of HER2-positive metastatic breast cancer with brain metastases, in which over 100 cycles of T-DM1 (107 cycles) were administered for more than 6 years, with durable intracranial and systemic disease stability and acceptable long-term tolerability.

## 2 Case description

In 2015, a 39-year-old woman was diagnosed with a HER2-positive invasive ductal carcinoma of the left breast with suspected axillary lymph node involvement. She received neoadjuvant chemotherapy with docetaxel and doxorubicin, followed by modified radical mastectomy in 2016. Pathological evaluation confirmed an invasive ductal carcinoma, staged as pT3N1aM0, with HER2 overexpression. The patient subsequently underwent adjuvant radiotherapy and trastuzumab-based systemic therapy in 2016.

In 2017, metastatic recurrence involving the lungs and bones was identified on chest computed tomography (CT) and a bone scan. Systemic therapy with docetaxel, trastuzumab, and pertuzumab was initiated; however, subsequent imaging revealed disease progression. Denosumab was added to manage the bone metastases.

**Figure 1:**
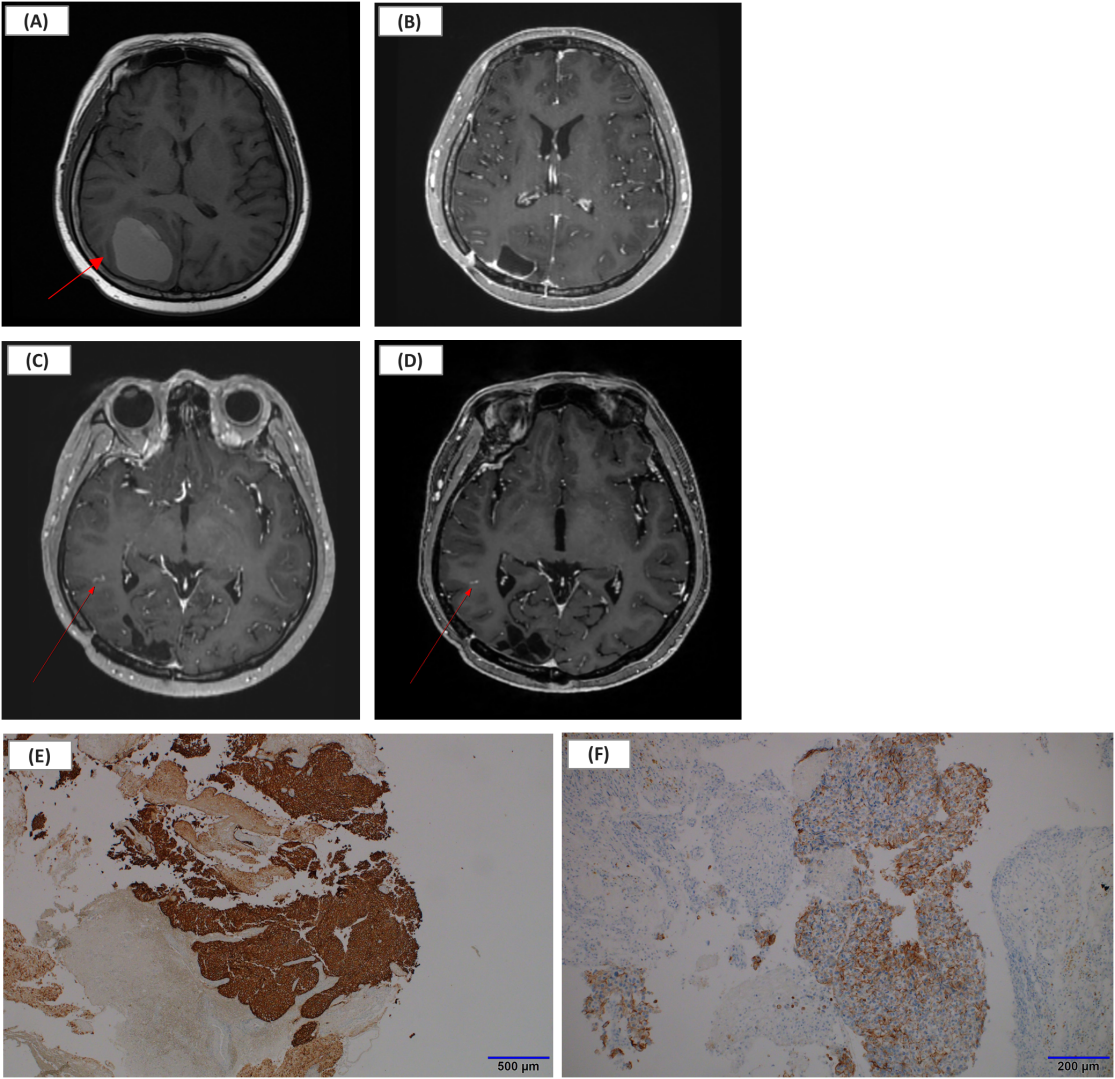
Serial brain MRI findings and histopathologic confirmation of brain metastasis during long-term T-DM1 therapy. **(A)** Representative axial contrast-enhanced T1-weighted brain MRI in 2019 demonstrating a dominant intracranial metastatic lesion (arrow) among multiple lesions, subsequently resected via craniotomy. **(B)** Follow-up MRI after craniotomy and gamma knife stereotactic radiosurgery showing stable treated lesions without new metastases during long-term T-DM1 therapy. **(C)** Brain MRI in January 2025 demonstrating a radiographically suspicious lesion (arrow), treated preemptively with stereotactic radiosurgery. **(D)** Most recent brain MRI in December 2025 showing no evidence of intracranial progression or new enhancing metastatic lesions. **(E)** HER2 immunohistochemistry showing strong membranous expression in metastatic tumor cells (×4; scale bar = 500 μm). **(F)** Cytokeratin (CK) immunohistochemistry confirming metastatic carcinoma cells (×10; scale bar = 200 μm). CK, cytokeratin; HER2, human epidermal growth factor receptor 2; MRI, magnetic resonance imaging; T-DM1, trastuzumab emtansine.

In 2019, brain metastases were identified through routine surveillance imaging. The patient underwent a craniotomy for a symptomatic intracranial lesion measuring 1.2 cm (**Figure 1A**). Histopathological analysis of the resected brain lesion confirmed a metastatic carcinoma with strong HER2 (3+) expression. Histopathological analysis of the resected brain lesion confirmed a metastatic carcinoma with strong HER2 (3+) expression. Immunohistochemical analysis of the primary breast tumor demonstrated estrogen receptor (ER) negative and progesterone receptor (PR) negative. The resected brain metastasis showed concordant ER and PR status, consistent with the primary tumor.

Gamma knife radiosurgery was subsequently performed for seven additional intracranial lesions to achieve local control.

**Figure 2:**
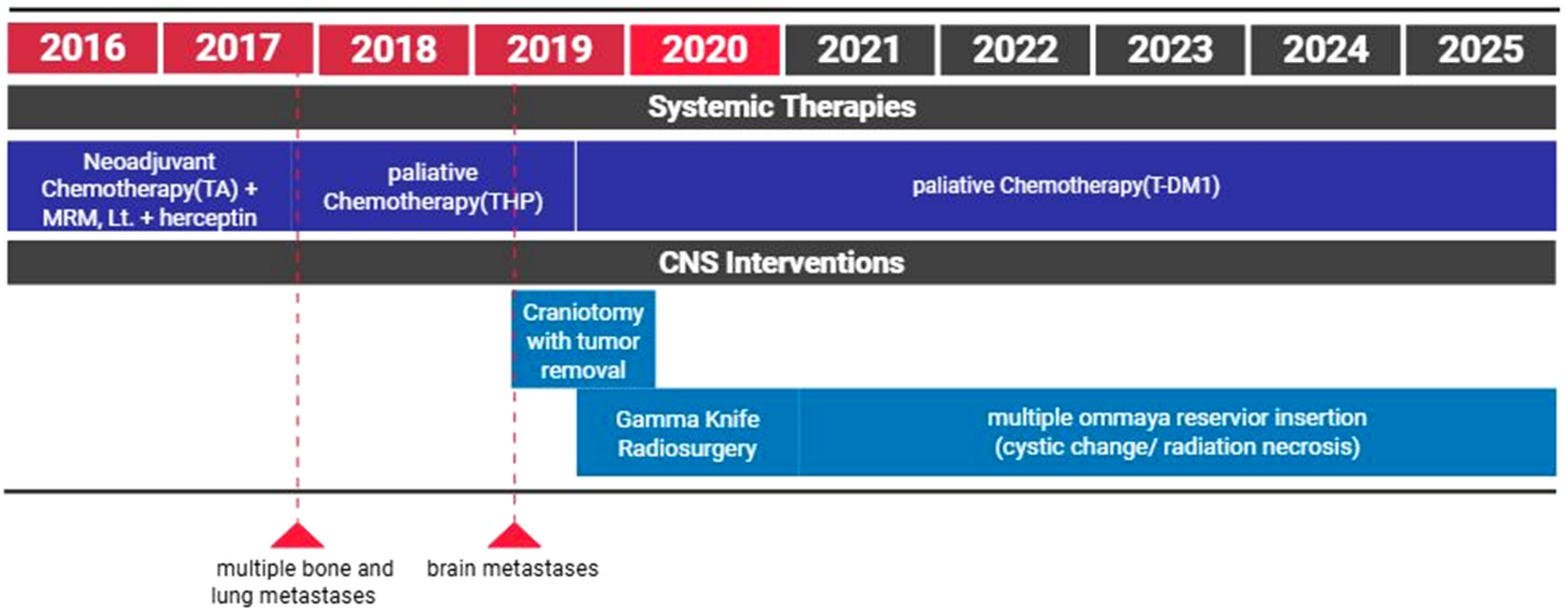
Schematic timeline of treatment course. Systemic therapies included neoadjuvant chemotherapy followed by surgery, palliative chemotherapy with THP, and prolonged trastuzumab emtansine (T-DM1) therapy. CNS-directed interventions consisted of craniotomy with tumor removal, gamma knife radiosurgery, and subsequent Ommaya reservoir insertions for management of cystic changes related to radiation necrosis. Red arrows indicate the timing of systemic disease progression and detection of brain metastases. TA, taxane–anthracycline–based chemotherapy; MRM, modified radical mastectomy; THP, docetaxel, trastuzumab, and pertuzumab; T-DM1, trastuzumab emtansine; CNS, central nervous system.

After local CNS-directed therapy, systemic T-DM1 treatment was initiated on May 17, 2019. During the disease course, the patient developed recurrent brain metastases on multiple occasions, requiring one craniotomy and repeated stereotactic radiosurgery for a total of eight intracranial lesions, as well as subsequent neurosurgical interventions including Ommaya reservoir placement for radiation necrosis–related cystic changes. T-DM1, an antibody-drug conjugate(ADC), was administered at 3.6 mg/kg every 3 weeks until November 6, 2025, for a total of 107 cycles. The patient had an Eastern Cooperative Oncology Group (ECOG) performance status of 0 at the initiation of T-DM1 treatment and remained clinically stable with an ECOG performance status of approximately 1 at the most recent follow-up. The overall treatment course, including systemic therapy, CNS-directed interventions, and key imaging milestones, is summarized in **Figure 2**.

The patient tolerated therapy well. Serial radiologic evaluations, including brain magnetic resonance imaging and systemic imaging such as chest computed tomography, were performed every 3 to 4 months and demonstrated sustained stabilization of both systemic and intracranial disease without the emergence of new metastatic lesions (**Figure 1B**). Response assessment during follow-up was based on routine clinical radiologic interpretation by experienced radiologists rather than formal application of standardized response criteria such as RECIST for systemic disease or RANO-BM for CNS lesions.

In January 2025, a radiographically suspicious intracranial lesion was identified and treated preemptively with gamma knife radiosurgery (**Figure 1C**), and no subsequent intracranial progression was observed on follow-up brain MRI through October 2025 (**Figure 1D**).

During the long-term follow-up, intermittent cystic chserianges at prior radiosurgery sites were observed and interpreted as radiation necrosis. When clinically indicated, these findings were managed conservatively, including Ommaya reservoir placement. Mild intermittent elevations in total bilirubin levels occurred during treatment, with a peak value of 2.11 mg/dL in February 2025, consistent with Common Terminology Criteria for Adverse Events (CTCAE) grade 1 toxicity; no dose modification was required. Cardiac function was closely monitored throughout treatment. The left ventricular ejection fraction was 67% in January 2016, reached a nadir of 53% in February 2022, and measured 59% in December 2025. A decline in LVEF to 53% was observed during routine surveillance in February 2022; this reduction was not accompanied by clinical symptoms of heart failure and did not meet criteria for treatment interruption. Cardiac function was monitored regularly with transthoracic echocardiography at approximately 3- to 4-month intervals, with good adherence to the planned monitoring schedule. During the course of treatment, cardiology consultation was obtained on multiple occasions to assess the safety of continued systemic therapy, and continuation of T-DM1 was deemed appropriate based on multidisciplinary evaluation. At the most recent follow-up in November 2025, the patient maintained stable systemic and intracranial disease with a good performance status. The overall clinical course and treatment timeline are summarized in **Table 1**. Adverse events observed during long-term T-DM1 therapy and their management are summarized in **Table 2**.

**Table 1:** Clinical course and treatment timeline.

Date (Month/Year)	Disease status/Event	Intervention	Response/Outcome
Dec 2015	Primary diagnosis (left breast IDC, HER2+)	Neoadjuvant docetaxel + doxorubicin	–
Jun 2016	Surgery	Modified radical mastectomy	pT3N1aM0; HER2 overexpression
Jul 2016	Adjuvant therapy	Radiotherapy + trastuzumab-based therapy	Completed
Jul 2018	Systemic recurrence (lung and bone metastases; detected on chest CT and bone scintigraphy)	Docetaxel + trastuzumab + pertuzumab	Progression
2018–present	Bone management	Denosumab	Ongoing
Apr 2019	Brain metastases	Craniotomy (dominant 1.2-cm lesion)	Pathology: HER2 3+ confirmed
May 2019	Residual brain lesions	Gamma knife stereotactic radiosurgery (7 lesions)	Local control
May 2019	Start T-DM1	T-DM1 initiated (3.6 mg/kg every 3 weeks)	Durable systemic/intracranial disease stability
2019–2025	Surveillance	Brain MRI and systemic CT every 3–4 months	No new metastatic lesions
Jan 2025	Radiographically suspicious CNS lesion	Preemptive gamma knife stereotactic radiosurgery	Stable thereafter
Feb 2025	Peak bilirubin	Total bilirubin 2.11 mg/dL (CTCAE grade 1)	No dose modification
Jan 2016/Feb 2022/Dec 2025	Cardiac monitoring (LVEF)	67%/53%/59%	Preserved
Nov 2025	Most recent T-DM1 administration	Total 107 cycles completed	Stable disease maintained

CK, cytokeratin; CNS, central nervous system; CT, computed tomography; CTCAE, Common Terminology Criteria for Adverse Events; HER2, human epidermal growth factor receptor 2; LVEF, left ventricular ejection fraction; MRI, magnetic resonance imaging; T-DM1, ado-trastuzumab emtansine.

**Table 2:** Adverse events during long-term T-DM1 therapy.

Adverse event	CTCAE grade	Management
Hyperbilirubinemia	Grade 1	Observation; no dose modification
Decline in LVEF	Grade 1	Continued therapy with close echocardiographic monitoring
Fatigue	Grade 1	Supportive care

LVEF, left ventricular ejection fraction.

## 3 Discussion

Brain metastasis remains a clinically significant issue in patients with HER2-positive metastatic breast cancer, particularly in the context of long-term disease control. Although pivotal trials of HER2-targeted therapies have established their systemic efficacy, patients with active brain metastases have largely been excluded from these studies, limiting the available evidence regarding prolonged intracranial disease management (1). As systemic therapies improve survival outcomes, CNS disease progression has emerged as an increasingly relevant challenge during extended treatment (1).

T-DM1 is an established standard therapy for previously treated HER2-positive metastatic breast cancer based on the phase III EMILIA trial, although intracranial efficacy was not a primary endpoint in these studies (2, 3). However, both clinical trial data and real-world cohorts indicate that treatment duration is typically limited to less than one year, with discontinuation most commonly due to disease progression or adverse events (4). In a whole-of-population Australian cohort, the median duration of T-DM1 treatment in routine clinical practice was 6.5 months (interquartile range, 3.1–13.5), underscoring that long-term continuous administration extending beyond multiple years—and particularly beyond 100 cycles—is exceedingly rare (4).

The present case is notable for exceptionally prolonged T-DM1 administration—107 cycles over more than 6 years (May 2019 to November 2025)—with sustained control of both systemic and intracranial diseases and acceptable long-term tolerability. Serial imaging performed every 3 to 4 months demonstrated durable intracranial stability without the development of new lesions (**Figures 1B–D**). Notably, a radiographically suspicious lesion identified in January 2025 was preemptively treated with stereotactic radiosurgery (**Figure 1C**), and continued intracranial stability was observed until December 2025 (**Figure 1D**). Rather than adhering to predefined limits of treatment duration, this case emphasizes the importance of individualized treatment decisions guided by long-term disease control, tolerability, and patient performance status.

However, long-term continuation raises concerns regarding cumulative toxicity, particularly hepatotoxicity and cardiac dysfunction. In this patient, the toxicity remained manageable, with intermittent CTCAE grade 1 hyperbilirubinemia without the need for dose modification and preserved cardiac function on longitudinal monitoring. In addition, this case highlights the importance of distinguishing intracranial tumor progression from radiation necrosis following stereotactic radiosurgery, as cystic changes at the treated sites were managed conservatively, including Ommaya reservoir placement, when clinically indicated.

At our institution, management of brain metastases typically involves a multidisciplinary approach incorporating neurosurgery, radiation oncology, and medical oncology. Surgical resection or stereotactic radiosurgery is considered for limited intracranial disease, followed by close radiologic surveillance in conjunction with systemic therapy.

Importantly, the sustained intracranial stability observed in this patient should be interpreted as the result of a combined modality approach, incorporating both repeated CNS-directed local therapies and systemic treatment, rather than the effect of T-DM1 alone.

The therapeutic landscape for HER2-positive metastatic breast cancer has evolved substantially with the introduction of newer HER2-targeted agents demonstrating clinically meaningful CNS activity, including tucatinib-based regimens and trastuzumab deruxtecan. In the HER2CLIMB trial, the addition of tucatinib to trastuzumab and capecitabine significantly improved intracranial disease control and overall survival in patients with brain metastases, while trastuzumab deruxtecan has also shown promising systemic and intracranial activity in more recent studies (5–7).

At the time of T-DM1 initiation in 2019, alternative HER2-targeted agents with proven CNS activity were not widely available. Although current treatment algorithms may favor alternative sequencing, this case remains clinically informative by illustrating that prolonged continuation of an effective and well-tolerated therapy may be reasonable in selected patients achieving sustained disease stability.

The decision to continue T-DM1 for more than six years was based on a combination of clinical and patient-centered factors. At the time of treatment initiation in 2019, alternative HER2-targeted agents with proven CNS activity were not widely available, and T-DM1 represented an appropriate standard-of-care option following prior HER2-directed therapies. During treatment, the patient achieved sustained systemic and intracranial disease stability with manageable toxicity, and expressed a clear preference to continue a well-tolerated regimen. In routine clinical practice, continuation of an effective therapy is generally favored in the absence of disease progression or unacceptable toxicity, and switching systemic treatment outside progression is uncommon. Ongoing multidisciplinary reassessment supported continued therapy throughout long-term follow-up.

From a biological perspective, preclinical studies have suggested that T-DM1 exerts antitumor activity within the brain microenvironment, providing biological plausibility for its potential contribution to CNS disease stability (8). While conclusions drawn from a single case are inherently limited, this report provides clinically meaningful insights into long-term management strategies for HER2-positive metastatic breast cancer with CNS involvement.

In summary, this case demonstrates that exceptionally prolonged administration of T-DM1, exceeding 100 cycles (107 cycles over more than 6 years), is feasible and may achieve durable control of brain metastases and systemic disease in carefully selected patients with HER2-positive metastatic breast cancer. In the absence of disease progression and manageable toxicity, continued T-DM1 therapy with close long-term surveillance may represent a reasonable individualized strategy.

## 4 Patient perspective

“After being diagnosed with brain metastases, I was worried about the possibility of further progression, particularly in the brain. Following surgery and radiosurgery, I started receiving T-DM1 treatment with regular imaging follow-up. Despite long-term therapy and ongoing hospital visits, I was able to maintain my daily activities without major side effects. Stable imaging results provided reassurance and allowed me to preserve a good quality of life.”

## Data Availability

The raw data supporting the conclusions of this article will be made available by the authors, without undue reservation.
